# BCG gone rogue: A case report of disseminated *Mycobacterium bovis*

**DOI:** 10.1016/j.idcr.2026.e02609

**Published:** 2026-05-14

**Authors:** Youssef Saklawi, Margaret Conrad, Mary Ann Kirkconnell Hall, Wassim Abdallah, Hady Samaha

**Affiliations:** a Division of Hospital Medicine, Emory University, 100 Woodruff Circle, Atlanta, GA 30322, USA; b Department of Medicine, Emory University, 1364 Clifton Rd NE, Atlanta, GA 30322, USA; c Division of Infectious Diseases, Emory University, 100 Woodruff Circle, Atlanta, GA 30322, USA

**Keywords:** *Mycobacterium bovis*, Bacillus Calmette–Guérin, Fever of unknown origin, Intravesical Bacillus Calmette–Guérin (BCG), Renal transplantation

## Abstract

**Introduction:**

Bacillus Calmette–Guérin (BCG) bladder instillation is an effective therapy for non–muscle-invasive bladder cancer. Disseminated *Mycobacterium bovis* infection is a rare but potentially life-threatening complication. Despite increasing use of intravesical BCG, evidence-based guidance regarding its administration in immunocompromised patients remains limited. Here, we report a case of disseminated *M. bovis* infection presenting as fever of unknown origin.

**Case presentation:**

A 59-year-old man presented to the hospital with fevers and chills. His past medical history was significant for renal transplantation with subsequent graft failure. He was maintained on tacrolimus 3 mg twice daily and prednisone 10 mg daily. He also had a history of bladder cancer treated with intravesical BCG immunotherapy 2 years prior to presentation. His hospital course was complicated by persistent undulating fevers. Computed tomography of the chest revealed a small cavitary lesion in the right upper lobe, and positron emission tomography imaging demonstrated increased uptake in the right iliopsoas muscle. A QuantiFERON Gold tuberculosis test was positive. Thirty days after presentation, acid-fast bacillus (AFB) blood cultures obtained at initial presentation, as well as AFB cultures from bronchoalveolar lavage and iliopsoas aspirate, grew pyrazinamide-resistant *Mycobacterium tuberculosis* complex. Subsequent sequencing identified *M. bovis*, consistent with disseminated infection related to prior intravesical BCG therapy.

**Conclusion:**

This case of fever of unknown origin was ultimately attributed to disseminated *M. bovis* infection, likely related to prior intravesical BCG therapy in an immunocompromised patient. Early recognition of this rare complication is critical, as disseminated *M. bovis* infection may present with severe systemic illness and multi-organ involvement.

## Highlights

• Late-onset hematogenous and pulmonary dissemination of *Mycobacterium bovis.*
• Immune reconstitution–like inflammation following withdrawal of tacrolimus.
• Hepatitis B surface antigenemia after vaccination in individual receiving dialysis.
• Positive QuantiFERON TB Gold Plus secondary to US strain of Bacillus Calmette–Guérin.

## Introduction

Bacillus Calmette–Guérin (BCG) is the first and only vaccine used to prevent *Mycobacterium tuberculosis* (MTB). It is made of live attenuated *Mycobacterium bovis*, a member of the *MTB* complex. Because of its immunomodulatory effects, including local effects when injected in the bladder, BCG is a therapy with proven efficacy in decreasing recurrence of non–muscle-invasive bladder cancer [1]. BCG therapy can be associated with many local and systemic complications that require special consideration, especially in immunocompromised patients [2]. Severe systemic complications of BCG therapy remain infrequent, with a rate of ≤ 1% [3]. Disseminated *Mycobacterium* infections (BCGosis) represent a serious complication of intravesical BCG therapy in immunocompromised patients. Such infections are characterized by widespread infection, usually involving the lungs, as well as other organs such as the liver, bone marrow, and bones [4].

Immunosuppressive therapies represent an important risk factor for developing BCGosis. While BCG for TB prevention is absolutely contraindicated in immune-compromised individuals, its use for bladder cancer is only “relatively contraindicated” [5]. Dissemination is driven by a weakening of the urothelial barrier facilitating the hematogenous spread of the attenuated version of *M. bovis* [6]. It remains an extremely rare presentation; however, symptoms of BCGosis can appear months to years after completion of intravesical therapy [4], [7].

## Case

### Patient information

We present the case of a 59-year-old male with history of lupus nephritis complicated by end-stage renal disease on hemodialysis using an arteriovenous graft; renal transplant with allograft failure; bladder cancer status post trans-urethral removal of bladder tumor; and intravesical BCG therapy 2 years prior. The patient had been undergoing a gradual outpatient taper of immunosuppression, including tacrolimus and prednisone, to reduce the risk of symptomatic graft rejection. The patient presented to the emergency department following 1 week of fevers, chills, and rigors. His symptoms were accompanied by worsening weakness, poor appetite, and asthenia.

### Clinical findings

**Fig. 1:**
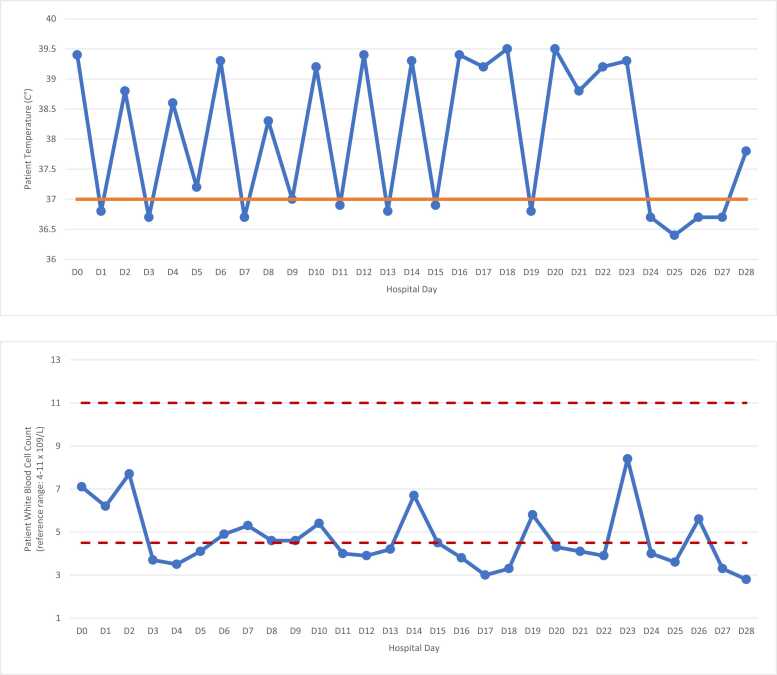
Fever curve and white blood cell count trend during hospitalization.

Vital signs and laboratory results on admission were notable for tachycardia and normocytic anemia, respectively, but no leukocytosis. Blood cultures collected showed growth of *Streptococcus sanguinis*, which cleared rapidly with ceftriaxone therapy. Tacrolimus was held in the setting of bacteremia. Despite negative blood cultures, the patient’s hospital stay was complicated by persistent intermittent high-grade fevers over 3 weeks. His peak temperature was 39.5° C. The patient’s fever curve and white blood cell count trend are illustrated in Fig. 1, and his hospital course is presented in Table 1.

**Table 1:** Case timeline.

**Hospital Day**	**Key Events and Findings**
Day 0	Immunocompromised patient presents with fever of unknown origin with evidence of bacteremia
Day 5	Persistent fever despite antibiotics; transthoracic and transesophageal echocardiography and arteriovenous graft ultrasound negative
Day 8	Viral and autoimmune work-up negative; AFB blood cultures obtained; tagged WBC scan showed pulmonary uptake
Day 18	Bronchioalveolar lavage and lung biopsy performed; bronchioalveolar lavage infectious work-up, including MTB PCR, was negative
Day 23	PET-CT revealed right iliopsoas abscess; CT-guided aspiration performed with initially negative cultures
Day 30	AFB blood culture from day 8 positive for MTB complex
Day 33	AFB cultures from lung and iliopsoas abscess positive for MTB complex
Day 34	Initiation of rifabutin, isoniazid, and ethambutol therapy with resolution of fever
Day 40	Genetic sequencing identified organism as BCG (modified *M. bovis*)

Abbreviations: AFB, acid-fast bacillus; BCG, Bacillus Calmette–Guérin; CT, computed tomography; MTB, *Mycobacterium tuberculosis*; PCR, polymerase chain reaction; PET-CT, positron emission tomography-computed tomography; WBC, white blood cells

### Diagnostic assessment

Evaluation of his left arteriovenous graft by vascular surgery and work-up including ultrasound was non-revealing. Transthoracic and transesophageal echocardiography did not show any vegetations. The patient’s daily fevers persisted.

**Fig. 2:**
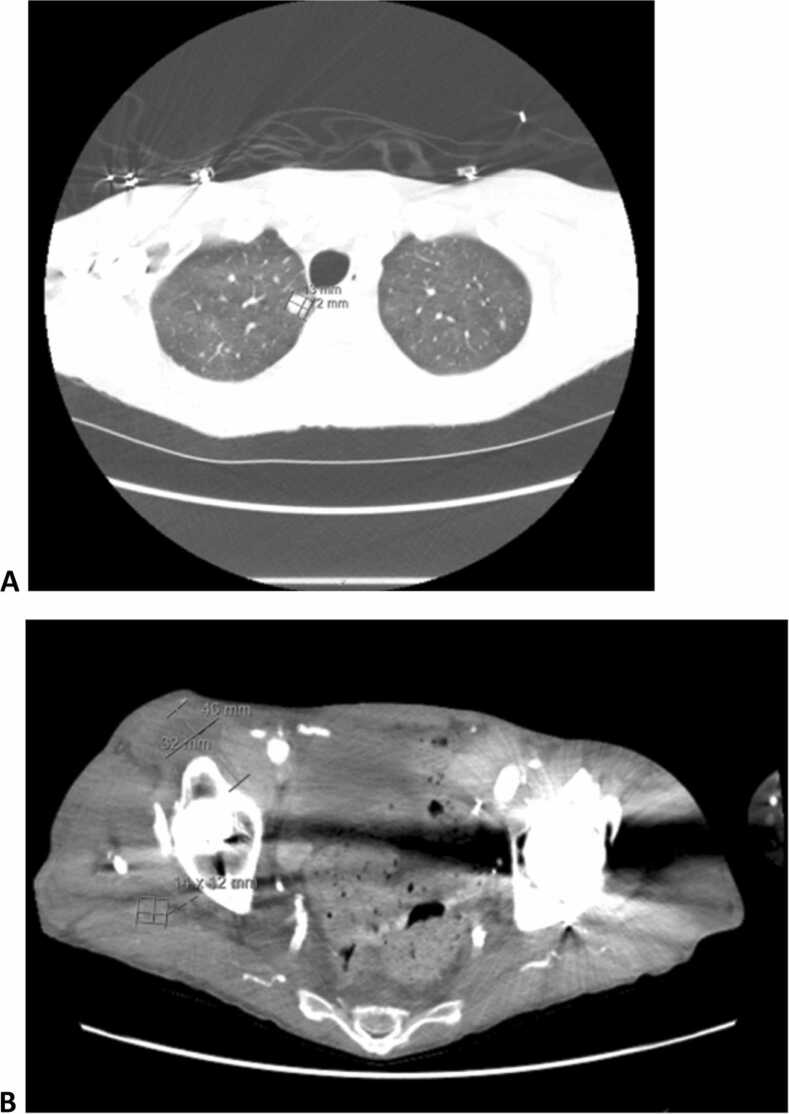
(A) CT Chest revealing right cavitary lung nodule. (B) CT Pelvis showing thick-walled fluid collections anterior and posterior to the right hip joint.

Further history revealed that the patient had always lived in Georgia, except for a short stint in Germany in the 1990s. He worked in a retail setting, and had no pets, outdoor exposures, or recent travel. He did not consume raw meat or unpasteurized dairy products. HIV and hepatitis tests were negative. Anti-nuclear antibody, antineutrophil cytoplasmic antibodies, anti-dsDNA, and complement levels were within normal limits. Serum Epstein–Barr virus (EBV) DNA was mildly positive; cytomegalovirus DNA was negative. Acid-fast bacillus (AFB) blood cultures were drawn. Serologies for *Brucella*, *Bartonella*, *Toxoplasma*, and *Coxiella* were negative. Low-level EBV viremia was detected on day 10 but subsequently down-trended on repeat testing. A tagged white blood cell scan was performed that did not show any uptake in the arteriovenous graft but did show uptake in the lungs. A computed tomography (CT) scan of the chest showed a small cavitary lesion in the right upper lobe and diffuse ground-glass opacities (Fig. 2). There was initial concern for EBV-associated post-transplant lymphoproliferative disorder, but the declining viral load and non-contributory imaging findings made this unlikely to be clinically relevant to the patient’s presentation.

Pulmonology was consulted and performed a bronchioalveolar lavage and biopsy. A pneumonia pathogen panel (by PCR) was negative for *Pneumocystis jirovecii* DNA, and bacterial, fungal, AFB, and *Legionella* cultures. Cytology was negative for malignancy. Urine *Histoplasma*, serum *Blastomyces*, and *Coccidioides* serologies were negative. A summary of the patient’s microbiology data collected during his hospital stay is highlighted in Table 2. A positron emission tomography (PET) scan was performed and showed increased uptake within the right iliopsoas muscle concerning for abscess. CT-guided aspiration of the abscess was performed. Fluid bacterial, fungal, and AFB cultures were initially negative, but a QuantiFERON (QFT) screen was positive. The patient continued to develop daily high-grade fevers. On day 30 of admission, an AFB blood culture collected on admission returned positive for MTB complex. Organism identification was initially performed using matrix-assisted laser desorption ionization–time of flight (MALDI-TOF), which identified the isolate as part of the MTB complex. The specimen was subsequently sent to the Georgia Department of Public Health laboratory for further phenotypic susceptibility testing and then to the Centers for Disease Control and Prevention for sequencing, which confirmed pyrazinamide-resistant *M. bovis* of BCG origin. Over the next few days, AFB cultures from lungs and iliopsoas abscess also grew *MTB* complex.

**Table 2:** Microbiology data collected during hospitalization.

Hospital Day	**Culture**	**Result**
Day 0	Blood culture	*Streptococcus sanguinis*
Day 3	Blood culture	Negative
Day 3	Hepatitis serologies	HBc Negative; HBsAg Positive; HBsAb Negative
Day 3	Stool culture	Negative
Day 8	HBV DNA	Undetected
Day 10	Blood culture	Negative
Day 10	Upper respiratory pathogen panel	Negative
Day 10	EBV PCR	Positive – 2900 IU/mL
Day 11	AFB blood culture	MTB complex, resulted on D30
Day 15	Blood culture	Negative
Day 17	EBV PCR	Positive – 1700 IU/mL
Day 17	BAL AFB culture	Negative
Day 17	BAL Fungal culture	Negative
Day 17	BAL *Legionella* culture	Negative
Day 17	BAL Respiratory culture	Negative
Day 17	(1,3)-Beta-D-Glucan	Positive – 154 pg/mL
Day 17	*Pneumocystis jirovecii* DNA	Negative
Day 17	MTB PCR	Negative
Day 25	Abscess AFB culture	MTB complex
Day 25	Abscess bacterial culture	Negative
Day 25	Abscess fungal culture	Negative
Day 38	(1,3)-Beta-D-Glucan	Negative - 50 pg/mL
Day 38	EBV PCR	< 300 IU/mL
Day 40	Wedge lung biopsy AFB culture	MTB complex

Abbreviations: AFB, acid-fast bacilli; BAL, bronchioalveolar lavage; EBV, Epstein Barr virus; MTB, *Mycobacterium tuberculosis*; PCR, polymerase chain reaction.

### Therapeutic intervention

The patient was started on isoniazid 300 mg daily with vitamin B6 25 mg daily, rifabutin 300 mg daily, and ethambutol 1200 mg every 48 h. Medication dosing was adjusted for end-stage renal disease, with a plan to complete at least 12 months of triple therapy. Pyrazinamide was avoided due to the pathogen’s intrinsic resistance profile.

### Follow-up and outcomes

The patient’s fevers subsided, the patient was safely discharged home. Culture sample sequencing revealed that it was consistent with *M. bovis*, confirmed as the strain used in his intravesical BCG therapy 2 years prior.

## Discussion

Our patient initially presented with fever, chills, and bacteremia, raising concern for an arteriovenous graft infection as the source of persistent fevers. However, despite appropriate antibiotic therapy and extensive evaluation, including vascular imaging and echocardiography, no evidence of graft-related infection was identified, and his fevers persisted for several weeks. This clinical course represents a classic presentation of fever of unknown origin secondary to BCGosis. Tuberculous mycobacterial cases range between 10% and 20% of cases of fever of unknown origin [7]. Table 2 summarizes a list of infectious differential diagnoses in cases of fever of unknown origin. Of note, serologic evaluation showed a positive hepatitis B surface antigen detected 5 days after vaccination with recombinant hepatitis B vaccine (Recombivax HB). Polymerase chain reaction testing for hepatitis B virus DNA was negative. Repeat testing demonstrated persistent surface antigen positivity 10 days after vaccination; the surface antigen became negative 28 days after vaccination. This transient antigenemia was attributed to recent vaccination [8] and was not considered to be the cause of the patient’s fever of unknown origin. Similarly, β-D-glucan was positive on day 10 but negative on repeat testing at day 38 and was not considered clinically significant and likely related to antimicrobial use. In fact, a study analyzing 35 antimicrobial drugs found that 25 of 35 (including 20 of 30 antibiotics and all 5 tested antifungals) contained sufficient β-D-glucan to trigger a positive test [9].

A history of prior intravesical BCG therapy, persistent fevers, and positive AFB cultures from multiple sites—including blood, iliopsoas abscess, and bronchoalveolar lavage—strongly supported the diagnosis of BCGosis.

BCG vaccination is absolutely contraindicated in individuals with impaired immune function, including those with HIV, congenital immunodeficiencies, hematologic malignancies, or those receiving immunosuppressive therapy. In one study, HIV-infected children had a several hundred-fold increased risk of disseminated BCG disease [10]. In contrast, intravesical BCG immunotherapy for bladder cancer is only relatively contraindicated in immunocompromised patients, and its use is generally considered on a case-by-case basis through shared decision-making. The patient’s chronic immunosuppression with tacrolimus and prednisone likely predisposed him to hematogenous dissemination. The temporal association between withdrawal of tacrolimus and the onset of persistent fevers raises the hypothesis of a possible immune reconstitution inflammatory syndrome–like response to underlying MTB.

Diagnosis of BCGosis is often challenging, as clinical manifestations vary widely depending on the organs involved, frequently resulting in delayed recognition. Bronchoalveolar lavage AFB smears and MTB PCR both have limited sensitivity (<10% and 38.8%, respectively) [11]. AFB cultures remain the gold standard despite prolonged turnaround times. Importantly, disseminated BCGosis reflects hematogenous spread in a miliary pattern and does not necessarily imply primary pulmonary infection, which may result in a low pulmonary inoculum (similar to miliary TB). If not promptly identified, disseminated infection may progress to extensive multi-organ involvement and severe sepsis [12]. In a systematic review, delayed BCGosis has been reported up to 24 months after intravesical administration. While the most common delayed presentations involve contiguous muscular or osteoarticular spread, late-onset hematogenous and pulmonary dissemination occurring 24 months after BCG exposure, as observed in this case, remains exceedingly rare [4]. Similar to other strains of *M. bovis*, BCG is intrinsically resistant to pyrazinamide. This is due to mutations in the pncA gene that impair pyrazinamidase activity required for drug activation [13]. Treatment consists of a multidrug regimen including isoniazid, ethambutol, and rifampin or rifabutin for a minimum duration of nine months, with extension guided by clinical response [14], [15].

Lastly, it is remarkable that our patient’s QFT was positive. While *M. bovis* causes positive QFT, BCG should not, as it lacks secreted T-cell antigens ESAT-6 and CFP-10 detected by the test [16]. A review of the literature shows that the BCG used in the USA (the TICE strain) is different than the BCG used for TB prevention internationally [17], [18], raising the hypothesis that the TICE strain may contain one or more of the antigens used in QFT testing. Additionally, the dose of BCG used for bladder cancer is significantly higher than the dose used for TB prevention [19], [20].

Fever of unknown origin in immunocompromised patients presents a broad differential, and delayed recognition of BCGosis may be mitigated through early consideration of next-generation sequencing or empiric anti-mycobacterial therapy.

## Funding

No funding was received for this work.

## CRediT authorship contribution statement

**Wassim Abdallah:** Writing – review & editing, Writing – original draft, Conceptualization. **Hady Samaha:** Writing – review & editing, Writing – original draft. **Margaret Conrad:** Writing – review & editing, Writing – original draft. **Mary Ann Kirkconnell Hall:** Writing – review & editing. **Saklawi, MD Youssef:** Writing – review & editing, Writing – original draft, Visualization, Validation, Supervision, Project administration, Conceptualization.

## Declaration of Competing Interest

The authors declare that they have no known competing financial interests or personal relationships that could have appeared to influence the work reported in this paper.
